# Effects of macitentan and selexipag across prognostic age groups in patients with pulmonary arterial hypertension

**DOI:** 10.1016/j.jhlto.2024.100197

**Published:** 2025-01-16

**Authors:** Richard Channick, Sarah Medrek, Marion Delcroix, Sean Gaine, Pavel Jansa, Irene Lang, Vallerie McLaughlin, Sanjay Mehta, Tomas Pulido, Bhagavatula Sastry, Rogerio Souza, Adam Torbicki, Carol Zhao, Paul Strachan, Peter Agron, Joseph Yen, Olivier Sitbon

**Affiliations:** a David Geffen School of Medicine at University of California Los Angeles, Los Angeles, CA; b University of New Mexico, Albuquerque, NM; c University Hospitals of Leuven, Leuven, Belgium; d National Pulmonary Hypertension Unit, Mater Misericordiae University Hospital, Dublin, Ireland; e Second Department of Internal Medicine–Department of Cardiovascular Medicine, First Faculty of Medicine, Charles University and General University Hospital, Prague, Czech Republic; f Department of Internal Medicine II, Cardiology and Center of Cardiovascular Medicine, Medical University of Vienna, Währinger Gürtel 18–20, 1090 Vienna, Austria; g University of Michigan, Ann Arbor, MI; h Respirology Division, Department of Medicine, Schulich School of Medicine and Dentistry, Western University, London, ON, Canada; i Clinical Research Department, National Heart Institute, Mexico City, Mexico; j CARE Hospitals, Hyderabad, India; k InCor, Hospital das Clínicas da Faculdade de Medicina da Universidade de São Paulo, São Paulo, Brazil; l Centre of Postgraduate Medical Education, European Health Centre, ECZ-Otwock, Poland; m Actelion Pharmaceuticals US, Inc., (a Johnson & Johnson Company), Titusville, NJ; n Actelion Pharmaceuticals US, Inc., (a Johnson & Johnson Company), Titusville, NJ; o Janssen Research and Development, LLC, Raritan, NJ; p Université Paris-Saclay, APHP, Hôpital Bicêtre, Le Kremlin-Bicêtre, France

**Keywords:** Pulmonary arterial hypertension, SERAPHIN, GRIPHON, Selexipag, Macitentan

## Abstract

**BACKGROUND:**

Age affects disease severity and patient outcomes in pulmonary arterial hypertension. This post-hoc analysis identified prognostic age groups and associated macitentan/selexipag treatment effects.

**METHODS:**

Randomized trials evaluated macitentan (SERAPHIN; NCT00660179) and selexipag (GRIPHON; NCT01106014) versus placebo (primary endpoint: time to morbidity/mortality [M/M]). This analysis defined age thresholds differentiating M/M risk in patients randomized to placebo (Cox regression determining treatment effect by age).

**RESULTS:**

Three age groups (< 35, 35–64, ≥ 65 years) showed good M/M risk discrimination (c-statistic 0.69, SERAPHIN; 0.66, GRIPHON). M/M risk was higher in placebo patients < 35 versus 35–64 years (SERAPHIN: hazard ratio [HR] 1.73, 95% confidence interval [CI] 1.10–2.72, *p* = 0.02; GRIPHON: HR 1.81, 95% CI 1.28–2.56, *p* < 0.001). M/M risk trended higher in patients ≥ 65 versus 35–64 years (SERAPHIN: HR 1.55, 95% CI 0.89–2.69, *p* = 0.12; GRIPHON (HR 1.08, 95% CI 0.75–1.55, *p* = 0.69). M/M risk was lower with macitentan/selexipag versus placebo: macitentan < 35 (HR 0.44, 95% CI 0.25–0.78; *p* = 0.005), 35–64 (HR 0.50, 95% CI 0.33–0.76; *p* < 0.001), ≥ 65 years (HR 0.69, 95% CI 0.30–1.58; *p* = 0.38); selexipag < 35 (HR 0.50, 95% CI 0.32–0.78; *p* = 0.002), 35–64 (HR 0.72, 95% CI 0.54–0.96; *p* = 0.03), ≥ 65 years (HR 0.55, 95% CI 0.33–0.91; *p* = 0.02). Adverse-event discontinuations were similar.

**CONCLUSIONS:**

The benefit (vs placebo) of macitentan/selexipag on reducing risk of M/M events was consistent across all ages, including the younger group where significant treatment effects were observed.

## Introduction

Pulmonary arterial hypertension (PAH) is a rare, often fatal, disease characterized by increased pulmonary artery pressure and increased pulmonary vascular resistance.^1^ Differences in disease severity, disease management, response to treatment, and survival have been observed between people of different ages.^2^, ^3^, ^4^, ^5^ Age-related differences in functional and hemodynamic assessments have also been reported.^6^ Utilizing different age thresholds, several research groups have identified that World Health Organization (WHO) functional class (FC) and 6-minute walk distance (6MWD) are more favorable in patients with PAH who are aged ≤ 65 years compared to older patients, despite higher mean pulmonary artery pressure and pulmonary vascular resistance in patients aged ≤ 65 years versus older patients.^2^, ^3^, ^4^, ^7^ Younger patients are likely to have fewer comorbidities than older patients, and the clinical attributes of PAH worsening may differ in younger patients.

Despite the differences in disease severity, disease management, and survival observed among patients with PAH of different ages, previous prespecified subgroup analyses of SERAPHIN and GRIPHON suggested a consistent treatment response with macitentan and selexipag, respectively, in patients aged ≥ 65 years and those aged 18–64 years.^8^, ^9^ The objectives of this post-hoc analysis of SERAPHIN and GRIPHON were to assess differences in PAH outcomes by age group using data from patients randomized to placebo in each study, and to determine whether treatment with macitentan (SERAPHIN) and selexipag (GRIPHON) improved outcomes for patients in particular age groups.

## Methods

### Design of original studies

The study designs and methods of SERAPHIN (NCT00660179)^10^ and GRIPHON (NCT01106014)^9^ have been published previously. Briefly, both trials were international, multicenter, double-blind, randomized, parallel-group, placebo-controlled, pivotal, event-driven, phase 3 studies with sufficiently similar methodologies to allow combination of outcomes data. Each protocol was approved by the institutional review board or independent ethics committee at each participating institution. All patients provided written informed consent.

SERAPHIN enrolled patients aged ≥ 12 years and GRIPHON enrolled patients aged 18–75 years. Both included patients who had idiopathic PAH or heritable PAH, or PAH associated with human immunodeficiency virus infection, drug use or toxin exposure, connective tissue disease, or repaired congenital systemic-to-pulmonary shunts.

In SERAPHIN, patients were randomized 1:1:1 to receive placebo, macitentan 3 mg once daily, or macitentan 10 mg once daily; the present analysis is limited to the macitentan 10 mg arm (i.e., the US Food and Drug Administration-approved dose). In GRIPHON, patients were randomized 1:1 to receive placebo or selexipag at an individualized maximum tolerated dose that was titrated starting at 200 µg twice daily to a maximum of 1600 µg twice daily. These studies enrolled in parallel, so it was not possible for patients in SERAPHIN (randomized to macitentan or placebo) to receive selexipag, or those in GRIPHON (randomized to selexipag or placebo) to receive macitentan during the studies.

At enrollment in SERAPHIN, patients were either under treatment with a stable dose for at least 3 months of oral phosphodiesterase type 5 inhibitors or oral or inhaled prostanoids, or were not under treatment with a PAH therapy; patients receiving intravenous or subcutaneous prostanoids were excluded. In GRIPHON, patients could either be receiving no treatment for PAH or could be receiving an endothelin receptor antagonist, a phosphodiesterase type 5 inhibitor, or both, at a dose that had been stable for ≥ 3 months; patients receiving prostacyclin analogs were excluded.

The current analysis included data for all patients randomized to 10 mg macitentan or placebo in SERAPHIN and all patients in GRIPHON.

### Outcome measures in original studies

As previously described in SERAPHIN^10^ and GRIPHON,^9^ the primary endpoints were time to morbidity/mortality (M/M), defined as time from treatment initiation to first M/M event (M/M definitions described in Table S1). Across the studies, PAH-related complications included disease progression or worsening of PAH or symptoms with or without hospitalization, need for additional PAH treatment, initiation of parenteral prostanoid therapy, long-term oxygen therapy, lung transplantation and balloon atrial septostomy. The main difference in the composite primary endpoint between the studies was inclusion of hospitalization for worsening PAH in GRIPHON but not in SERAPHIN. M/M events were adjudicated by a blinded critical-event committee. Prespecified secondary endpoints in both studies included change in 6MWD, change in NYHA/WHO FC from baseline to month 6, death due to PAH or hospitalization for worsening of PAH up to the end of the treatment period, and death from any cause up to the end of the study (see Table S1 for details). Safety endpoints included adverse events and laboratory abnormalities.

### Analysis of prognostic age groups in the placebo arms

For the present analysis, the placebo arms from SERAPHIN and GRIPHON were used to identify age thresholds differentiating M/M risk. Patients from each study were grouped by 5-year age increments. Age thresholds were identified using Kaplan–Meier estimates of the time to M/M events, hazard ratios (HRs; 95% confidence interval [CI]) derived by Cox proportional hazards models (adjusted for covariates at baseline: NYHA/WHO FC, PAH medication, geographical region, PAH etiology, sex, and race/ethnicity), and event rates at end of treatment, as well as c-statistics. The 36-month time point was chosen because at that point the event rate in the placebo arm of each study was > 50% (SERAPHIN 55%; GRIPHON 62%), allowing for more accurate event-rate estimates. The c-statistics were applied to determine the ability of the age threshold groupings to discriminate risk of an M/M event. The c-statistic describes concordance between the model prediction of an M/M event and the actual occurrence of an M/M event, with 0.5 indicating random concordance and 1 indicating perfect concordance.^11^

### Analysis of treatment effect according to prognostic age group

Following identification of the prognostic age groups in the above analysis, patient baseline demographic and clinical characteristics, collected per the individual study protocols, were analyzed by randomized treatment in the identified age groups. The treatment effects of macitentan or selexipag were then evaluated in the identified age groups using Cox regression models adjusted for sex, race/ethnicity, region, PAH etiology, NYHA/WHO FC, and PAH background therapy. Outcomes evaluated were time to the first M/M event, change from baseline in 6MWD at 6 months, mean number of all-cause hospitalizations per year, change from baseline in N-terminal prohormone B-type natriuretic peptide at 6 months, and adverse events.

For both determination of age thresholds and evaluation of the treatment effect, data from SERAPHIN and GRIPHON were analyzed separately. An interaction of treatment and age group was tested to assess potential heterogeneity effect. The analysis in this article is post hoc and exploratory in nature, and all *p*-values are nominal.

## Results

A total of 742 patients were enrolled in SERAPHIN (placebo, *n* = 250; macitentan 3 mg, *n* = 250; macitentan 10 mg, *n* = 242) and 1156 patients were enrolled in GRIPHON (placebo, *n* = 582; selexipag, *n* = 574).^9^, ^10^ All patients in the placebo and macitentan 10 mg arms of SERAPHIN (*n* = 492; of whom 13 [2.6%] were aged ≥ 12 years and < 18 years) and all patients in GRIPHON (*n* = 1156) were included in the present analysis.

### Prognostic age groups

**Figure 1:**
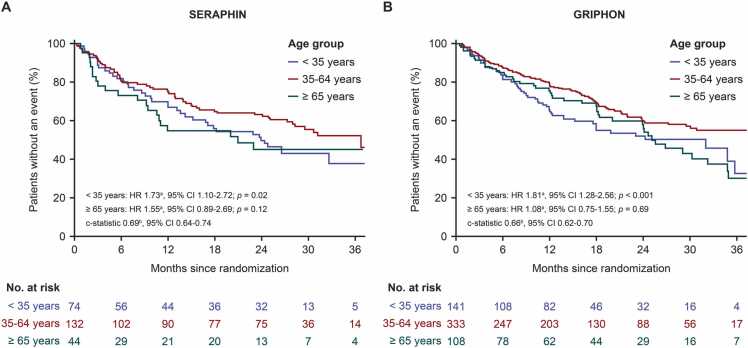
Identification of prognostic age groups. Kaplan-Meier curves of time to first morbidity/mortality event among patients randomized to placebo through to the end of study treatment (results shown up to 36 months for clarity) in (A) SERAPHIN (*n* = 250) and (B) GRIPHON (*n* = 582). ^a^Hazard ratio reflects comparison to ages 35–64 years (middle age group). Model adjusted for covariates at baseline: New York Heart Association/World Health Organization functional class, PAH medication, geographical regions, PAH etiology, sex, and race/ethnicity. ^b^The c-statistic measures the ability of the model to distinguish between groups. Scores range from 0.5 to 1.0; associated confidence intervals that include 0.5 are generally not considered significant. CI, confidence interval; HR, hazard ratio; PAH, pulmonary arterial hypertension.

The analysis used data from the placebo arms of each study. The median age in the SERAPHIN placebo arm was 46 years (interquartile range 32–61) and the median age in the GRIPHON placebo arm was 49 years (interquartile range 35–60). When assessing the relationship between age and M/M events, each data set yielded a non-linear curve with a greater likelihood of experiencing an M/M event in those aged < 35 years and ≥ 65 years (Figure S1A and S1B). Using c-statistics, three prognostic age groupings were identified with good discrimination of M/M risk: ≥ 18 and < 35 (younger age group), 35–64 (middle age group), and ≥ 65 (older age group) years (c-statistic 0.69, 95% CI 0.64–0.74 in SERAPHIN [Figure 1A]; c-statistic 0.66, 95% CI 0.62–0.70 in GRIPHON [Figure 1B]). In SERAPHIN, placebo-treated patients aged < 35 years had a 73% higher risk of experiencing an M/M event than placebo-treated patients aged 35–64 years (HR 1.73, 95% CI 1.10–2.72; *p* = 0.02 [Figure 1A]). Placebo-treated patients aged ≥ 65 years had a 55% higher risk of experiencing an M/M event compared to the middle age group (HR 1.55, 95% CI 0.89–2.69; *p* = 0.12 [Figure 1A]), but this difference was not statistically significant, potentially due to the small sample size in this group. In GRIPHON, placebo-treated patients aged < 35 years had an 81% higher risk of experiencing an M/M event compared to placebo-treated patients aged 35–64 years (HR 1.81, 95% CI 1.28–2.56; *p* <0.001 [Figure 1B]). Placebo-treated patients aged ≥ 65 years had a similar risk of experiencing an M/M event compared to the middle age group (HR 1.08, 95% CI 0.75–1.55); *p* = 0.69; however, the sample size was small (*n* = 27).

### Baseline characteristics according to prognostic age group

Baseline demographics and disease characteristics are shown by age group, according to randomized treatment, in Table 1 (SERAPHIN) and Table 2 (GRIPHON). In SERAPHIN, 27.6% of patients were aged < 35 years, 57.9% were aged 35–64 years, and 14.4% were aged ≥ 65 years; corresponding values in GRIPHON were 22.9%, 59.2%, and 17.9%, respectively. Some differences were observed among the age groups. The younger age group included a higher proportion of patients from Asia compared to the middle age group; and a higher proportion of patients from Western Europe and Israel were in the older age group. Body mass index was lower in the younger age group. Connective-tissue disease-associated PAH was more common among older patients, and congenital heart disease-associated PAH was more common among younger patients. A higher proportion of younger patients were in NYHA/WHO FC I/II and had longer 6MWD, whereas older patients were less likely to be in NYHA/WHO FC I/II and had shorter 6MWD. Comorbidities were numerically less common among younger patients. There were no notable differences in the background therapies received by patients in the different age groups in each study. Time since PAH diagnosis was shorter in younger patients in GRIPHON, but not in SERAPHIN.

**Table 1:** Baseline Characteristics by Identified Prognostic Age Group in SERAPHIN

	Placebo			Macitentan		
	Age < 35 Years (*n* = 74)	Age 35-64 Years (*n* = 132)	Age ≥ 65 Years (*n* = 44)	Age < 35 Years (*n* = 62)	Age 35-64 Years (*n* = 153)	Age ≥ 65 Years (*n* = 27)
Female, *n* (%)	51 (68.9)	100 (75.8)	33 (76.7)	49 (79.0)	123 (80.4)	22 (81.5)
*p*-value vs middle age group	0.29	—	0.90	0.82	—	0.90
Race/ethnicity, *n* (%)						
White	23 (31.1)	72 (54.5)	36 (83.7)	24 (38.7)	85 (55.6)	26 (96.3)
Asian	34 (45.9)	34 (25.8)	3 (7.0)	23 (37.1)	41 (26.8)	1 (3.7)
Hispanic	15 (20.3)	19 (14.4)	3 (7.0)	12 (19.4)	23 (15.0)	0
Black	2 (2.7)	5 (3.8)	1 (2.3)	3 (4.8)	3 (2.0)	0
Other	0	2 (1.5)	0	0	1 (0.7)	0
*p*-value vs middle age group	0.008	—	0.02	0.18	—	0.003
Geographical region, *n* (%)						
Asia	33 (44.6)	31 (23.5)	4 (9.3)	22 (35.5)	44 (28.8)	2 (7.4)
Eastern Europe/Turkey	20 (27.0)	34 (25.8)	5 (11.6)	16 (25.8)	39 (25.5)	7 (25.9)
Latin America	14 (18.9)	23 (17.4)	5 (11.6)	14 (22.6)	25 (16.3)	2 (7.4)
Western Europe/Israel	5 (6.8)	24 (18.2)	21 (48.8)	7 (11.3)	27 (17.6)	14 (51.9)
North America	2 (2.7)	20 (15.2)	8 (18.6)	3 (4.8)	18 (11.8)	2 (7.4)
*p*-value vs middle age group	0.001	—	< 0.001	0.30	—	0.002
BMI, median (IQR), kg/m^2^	22.5 (19.8−25.6)	24.9 (22.1−29.3)	26.1 (23.7−28.9)	22.1 (19.6−24.5)	25.2 (22.3−29.8)	25.5 (23.3−29.8)
*p*-value vs middle age group	< 0.001	—	0.51	< 0.001	—	0.69
Time since PAH diagnosis, median (IQR), years	1.5 (0.5−3.7)	1.3 (0.5−3.8)	1.0 (0.4−2.0)	1.2 (0.4−3.4)	1.6 (0.6−3.1)	0.4 (0.3−1.2)
*p*-value vs middle age group	0.65	—	0.20	0.92	—	0.12
PAH etiology, *n* (%)						
Idiopathic, heritable, drug or toxin induced, HIV-associated	42 (56.8)	75 (57.7)	20 (46.5)	40 (64.5)	89 (58.6)	16 (59.3)
Connective tissue disease-associated	16 (21.6)	43 (33.1)	22 (51.2)	13 (21.0)	51 (33.6)	9 (33.3)
Congenital heart disease-associated	16 (21.6)	12 (9.2)	1 (2.3)	9 (14.5)	12 (7.9)	2 (7.4)
*p*-value vs middle age group	0.02	—	0.06	0.10	—	> 0.99
NYHA/WHO FC, *n* (%)^a^						
I/II	51 (68.9)	65 (49.2)	13 (30.2)	35 (56.5)	76 (49.7)	10 (37.0)
III/IV	23 (31.1)	67 (50.8)	30 (69.8)	27 (43.5)	77 (50.3)	17 (63.0)
*p*-value vs middle age group	0.006	—	0.03	0.37	—	0.23
6MWD, median (IQR), meters	367.5 (286.0−435.0)	366.5 (300.0−440.0)	306.5 (223.5−367.0)	395.0 (328.0−448.0)	360.0 (300.0−434.0)	318.0 (251.0−396.0)
*p*-value vs middle age group	0.91	—	< 0.001	0.14	—	0.02
NT-proBNP, median (IQR), ng/L	673.0 (456.7−1158.1)	720.1 (527.3−1242.1)	849.2 (616.6−1481.9)	722.1 (521.0−1194.7)	777.4 (546.5−1373.8)	952.74 (672.2−1730.8)
*p*-value vs middle age group	0.40	—	0.20	0.75	—	0.16
Received background PAH therapy, *n* (%)	49 (66.2)	81 (61.4)	24 (54.5)	42 (67.7)	97 (63.4)	15 (55.6)
PDE5 inhibitor	47 (63.5)	79 (59.8)	24 (54.5)	42 (67.7)	94 (61.4)	14 (51.9)
Prostacyclin analog	4 (5.4)	2 (1.5)	1 (2.3)	3 (4.8)	12 (7.8)	1 (3.7)
Did not receive background PAH therapy, *n* (%)	25 (33.8)	51 (38.6)	20 (45.5)	20 (32.3)	56 (36.6)	12 (44.4)
Any comorbidity, *n* (%)	65 (87.8)	126 (95.5)	42 (95.5)	58 (93.5)	147 (96.1)	27 (100.0)
Total comorbidities in medical history, *n*	315	891	424	237	1180	215
Comorbidities in medical history occurring in > 20% of patients in any group, *n* (%)						
Hypertension	3 (4.1)	42 (31.8)	25 (56.8)	6 (9.7)	58 (37.9)	22 (81.5)
Right ventricular failure	32 (43.2)	47 (35.6)	12 (27.3)	17 (27.4)	59 (38.6)	10 (37.0)
Scleroderma	4 (5.4)	16 (12.1)	12 (27.3)	1 (1.6)	28 (18.3)	9 (33.3)
Raynaud’s phenomenon	1 (1.4)	11 (8.3)	12 (27.3)	1 (1.6)	15 (9.8)	3 (11.1)
Hypothyroidism	4 (5.4)	10 (7.6)	9 (20.5)	2 (3.2)	17 (11.1)	4 (14.8)
Atrial fibrillation	0	0	0	0	6 (3.9)	6 (22.2)
Osteoporosis	0	0	0	0	4 (2.6)	7 (25.9)

6MWD, 6-minute walk distance; BMI, body mass index; HIV, human immunodeficiency virus; IQR, interquartile range; NT-proBNP, N-terminal prohormone B-type natriuretic peptide; NYHA/WHO FC, New York Heart Association/World Health Organization functional class; PAH, pulmonary arterial hypertension; PDE5, phosphodiesterase type 5.

a Overall, only one patient was NYHA/WHO FC I and 14 were NYHA/WHO FC IV.

**Table 2:** Baseline Characteristics by Identified Prognostic Age Group in GRIPHON

	Placebo			Selexipag		
	Age < 35 Years (*n* = 141)	Age 35-64 Years (*n* = 333)	Age ≥ 65 Years (*n* = 108)	Age < 35 Years (*n* = 124)	Age 35-64 Years (*n* = 351)	Age ≥ 65 Years (*n* = 99)
Female, *n* (%)	109 (77.3)	280 (84.1)	77 (71.3)	92 (74.2)	287 (81.8)	78 (78.8)
*p*-value vs middle age group	0.08	—	0.003	0.07	—	0.50
Race/ethnicity, *n* (%)						
White	56 (39.7)	222 (66.7)	97 (89.8)	54 (43.5)	232 (66.1)	90 (90.9)
Asian	62 (44.0)	53 (15.9)	5 (4.6)	54 (43.5)	70 (19.9)	1 (1.0)
Hispanic	20 (14.2)	40 (12.0)	3 (2.8)	15 (12.1)	31 (8.8)	5 (5.1)
Black	1 (0.7)	11 (3.3)	2 (1.9)	0	11 (3.1)	2 (2.0)
Other	2 (1.4)	7 (2.1)	1 (0.9)	1 (0.8)	7 (2.0)	1 (1.0)
*p*-value vs middle age group	< 0.001	—	< 0.001	< 0.001	—	< 0.001
Geographical region, *n* (%)						
Asian	60 (42.6)	49 (14.7)	4 (3.7)	51 (41.1)	63 (17.9)	1 (1.0)
Eastern Europe	39 (27.7)	88 (26.4)	28 (25.9)	34 (27.4)	98 (27.9)	17 (17.2)
Latin America	18 (12.8)	34 (10.2)	4 (3.7)	12 (9.7)	36 (10.3)	6 (6.1)
North America	7 (5.0)	70 (21.0)	21 (19.4)	14 (11.3)	53 (15.1)	28 (28.3)
Western Europe/ Australia	17 (12.1)	92 (27.6)	51 (47.2)	13 (10.5)	101 (28.8)	47 (47.5)
*p*-value vs middle age group	< 0.001	—	< 0.001	< 0.001	—	< 0.001
BMI, median (IQR), kg/m^2^	21.8 (19.9−25.8)	26.4 (23.0−31.2)	26.7 (24.2−31.0)	21.8 (19.7−25.9)	26.2 (22.6−30.8)	27.6 (24.8−32.0)
*p*-value vs middle age group	< 0.001	—	0.94	< 0.001	—	0.15
Time since PAH diagnosis, median (IQR), years	0.6 (0.1−1.9)	1.2 (0.3−4.2)	1.1 (0.4−2.3)	0.5 (0.1−2.0)	1.0 (0.3−3.4)	1.4 (0.5−3.2)
*p*-value vs middle age group	0.002	—	0.01	0.02	—	0.81
Etiology of PAH, *n* (%)						
Idiopathic, heritable, drug or toxin induced, HIV-associated	91 (64.5)	216 (64.9)	58 (53.7)	74 (59.7)	215 (61.3)	58 (58.6)
Connective tissue disease-associated	29 (20.6)	91 (27.3)	47 (43.5)	24 (19.4)	106 (30.2)	37 (37.4)
Congenital heart disease-associated	21 (14.9)	26 (7.8)	3 (2.8)	26 (21.0)	30 (8.5)	4 (4.0)
*p*-value vs middle age group	0.03	—	0.003	< 0.001	—	0.18
NYHA/WHO FC, n (%)^a^						
I/II	80 (56.7)	148 (44.4)	32 (29.6)	76 (61.3)	176 (50.1)	26 (26.3)
III/IV	61 (43.3)	185 (55.6)	76 (70.4)	48 (38.7)	175 (49.9)	73 (73.7)
*p*-value vs middle age group	0.01	—	0.007	0.03	—	< 0.001
6MWD, median (IQR), meters	387.0 (357.0−426.0)	370.0 (300.0−417.0)	317.5 (240.0−375.0)	394.5 (346.5−428.0)	382.0 (330.0−420.0)	320.0 (259.0−369.0)
*p*-value vs middle age group	< 0.001	—	< 0.001	0.07	—	< 0.001
NT-proBNP, median (IQR), ng/L	520.0 (159.0−1802.0)	504.0 (179.0−1563.0)	996.0 (290.0−2397.0)	488.0 (152.0−1498.0)	512.5 (166.5−1394.0)	815.5 (347.0−1761.0)
*p*-value vs middle age group	0.60	—	0.07	0.33	—	0.008
Received background PAH therapy, *n* (%)	103 (73.0)	261 (78.4)	94 (87.0)	94 (75.8)	279 (79.5)	89 (89.9)
ERA plus PDE5 inhibitor	31 (22.0)	125 (37.5)	41 (38.0)	31 (25.0)	109 (31.1)	39 (39.4)
ERA monotherapy	13 (9.2)	43 (12.9)	20 (18.5)	18 (14.5)	59 (16.8)	17 (17.2)
PDE5 inhibitor monotherapy	59 (41.8)	93 (27.9)	33 (30.6)	45 (36.3)	111 (31.6)	33 (33.3)
Did not receive background PAH therapy, *n* (%)	38 (27.0)	72 (21.6)	14 (13.0)	30 (24.2)	72 (20.5)	10 (10.1)
Any comorbidity, *n* (%)	133 (94.3)	323 (97.0)	108 (100.0)	110 (88.7)	338 (96.3)	99 (100.0)
Total comorbidities in medical history, *n*	665	2849	1137	577	2647	1161
Comorbidities in medical history occurring in > 20% of patients in any group, *n* (%)						
Hypertension	8 (5.7)	97 (29.1)	65 (60.2)	4 (3.2)	117 (33.3)	62 (62.6)
Systemic sclerosis	5 (3.5)	52 (15.6)	37 (34.3)	2 (1.6)	49 (14.0)	25 (25.3)
Gastroesophageal reflex disease	5 (3.5)	51 (15.3)	28 (25.9)	6 (4.8)	50 (14.2)	28 (28.3)
Hypothyroidism	13 (9.2)	37 (11.1)	18 (16.7)	6 (4.8)	40 (11.4)	22 (22.2)

6MWD, 6-minute walk distance; BMI, body mass index; ERA, endothelin receptor antagonist; HIV, human immunodeficiency virus; IQR, interquartile range; NT-proBNP, N-terminal prohormone B-type natriuretic peptide; PAH, pulmonary arterial hypertension; PDE5, phosphodiesterase type 5; NYHA/WHO FC, New York Heart Association/World Health Organization functional class.

a Overall, only nine patients were NYHA/WHO FC I and 11 were NYHA/WHO FC IV.

### Treatment effect according to prognostic age group

**Figure 2:**
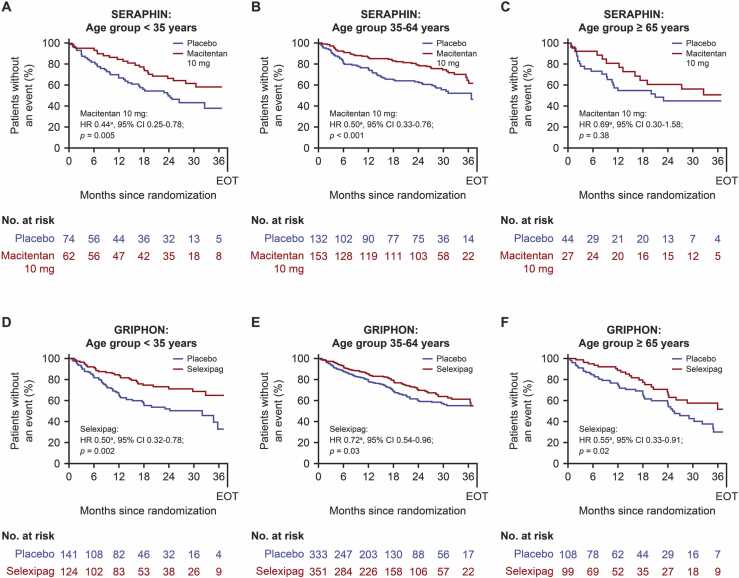
Evaluation of treatment effect in identified prognostic age groups. Kaplan–Meier curves of time to first morbidity/mortality event among patients in SERAPHIN aged (A) < 35 years, (B) 35–64 years, and (C) ≥ 65 years; and patients in GRIPHON aged (D) < 35 years, (E) 35–64 years, and (F) ≥ 65 years. ^a^Model adjusted for covariates at baseline: New York Heart Association/World Health Organization functional class, PAH medication, geographical regions, PAH etiology, sex, and race/ethnicity. CI, confidence interval; HR, hazard ratio; PAH, pulmonary arterial hypertension.

Interaction tests showed a lack of heterogeneity in treatment effect between age groups (*p* = 0.97 for SERAPHIN; *p* = 0.17 for GRIPHON). The treatment effects of macitentan and selexipag on reducing the risk of an M/M event versus placebo were evident and consistent across all age groups. Macitentan-treated patients aged < 35 years had a 56% relative risk reduction (HR 0.44 vs placebo, 95% CI 0.25–0.78; *p* = 0.005 [Figure 2A]), patients aged 35–64 years had a 50% relative risk reduction (HR 0.50 vs placebo, 95% CI 0.33–0.76; *p* < 0.001 [Figure 2B]), and patients aged ≥ 65 years had a 31% relative risk reduction (HR 0.69 vs placebo, 95% CI 0.30–1.58; *p* = 0.38 [Figure 2C]), though this was not statistically significant due to small sample sizes in this age group yielding wide 95% CIs. Selexipag-treated patients aged < 35 years had a 50% relative risk reduction (HR 0.50 vs placebo, 95% CI 0.32–0.78; *p* = 0.002 [Figure 2D]), patients aged 35–64 years had a 28% relative risk reduction (HR 0.72 vs placebo, 95% CI 0.54–0.96; *p* = 0.03 [Figure 2E]), and patients aged ≥ 65 years had a 45% relative risk reduction (HR 0.55 vs placebo, 95% CI 0.33–0.91; *p* = 0.02 [Figure 2F]).

The 6MWD increased from baseline to 6 months compared to placebo across most age groups treated with macitentan or selexipag, with the exception of patients aged ≥ 65 years receiving selexipag (Table 3). The mean rate of annualized all-cause hospitalizations was lower in patients treated with macitentan or selexipag versus placebo across all age groups (Table 4). Median N-terminal prohormone B-type natriuretic peptide levels generally decreased from baseline to 6 months and were compared to placebo across most age groups in patients treated with macitentan or selexipag (Table 5), whereas median changes from baseline to 6 months among treated patients were greatest in the middle age group receiving macitentan and in the older age group receiving selexipag.

**Table 3:** Evaluation of Treatment Effect in Identified Prognostic Age Groups: Change in 6-Minute Walk Distance in SERAPHIN and GRIPHON^a^

Seraphin	Age < 35 Years		Age 35-64 Years		Age ≥ 65 Years	
	Placebo (*n* = 74)	Macitentan (*n* = 62)	Placebo (*n* = 132)	Macitentan (*n* = 153)	Placebo (*n* = 44)	Macitentan (*n* = 27)
Baseline, *n*	74	62	132	153	43	27
6MWD, at baseline median (Q1, Q3), meters	367.5 (286.0, 435.0)	395 (328.0, 448.0)	366.5 (300.0, 440.0)	360 (300.0, 434.0)	309 (227.0, 368.0)	318 (251.0, 396.0)
Change from baseline to week 26, *n*	57	55	111	133	29	24
6MWD, median change (Q1, Q3), meters	23 (0.0, 72)	27 (−8, 75)	6 (−26, 45)	20 (−6, 61)	−2 (−20, 35)	7.5 (−16, 39)
*p*-value vs middle age group	0.025	0.86	Ref	Ref	0.87	0.57

GRIPHON	Age < 35 years		Age 35−64 years		Age ≥ 65 years	
	Placebo (*n* = 141)	Selexipag (*n* = 124)	Placebo (*n* = 333)	Selexipag (*n* = 351)	Placebo (*n* = 108)	Selexipag (*n* = 99)

Baseline, n	141	124	333	351	108	99
6MWD at baseline, median (Q1, Q3), meters	387.0 (357.0, 426.0)	394.5 (346.5, 428.0)	370.0 (300.0, 417.0)	382.0 (330.0, 420.0)	317.5 (240.0, 375.0)	320.0 (259.0, 369.0)
Change from baseline to month 6, *n*	141	124	333	351	108	99
6MWD, median change (Q1, Q3), meters	2.0 (−103.0, 42.0)	17.5 (23.0, 37.5)	−9.0 (−135.0, 24.0)	5.0 (−55.0, 38.0)	−19.0 (−169.5, 13.0)	−26.0 (−180.0, 15.0)
*p*-value vs middle age group	0.26	0.22	Ref	Ref	0.53	0.01

6MWD, 6-minute walk distance; CI, confidence interval; Q, quarter; Ref, reference value.

a These data need to be interpreted within the context of potential imbalances in baseline characteristics (Table 1 and Table 2) between the treatment and placebo groups in relation to the reference group (age 35–64 years). Size of treatment effect in the SERAPHIN and GRIPHON studies should also be considered when interpreting the data. In SERAPHIN the treatment effect was 22 m (97.5% CI 3.2–40.8, *p* = 0.008) with 10 mg macitentan versus placebo.^10^ In GRIPHON, the treatment effect was 12 m (99% CI 1–24, *p* = 0.003).^9^ In addition, sample size for the age groups in the current analysis should also be considered.

**Table 4:** Evaluation of Treatment Effect in Prognostic Age Groups: Annualized Hospitalizations in SERAPHIN and GRIPHON

	Age < 35 Years		Age 35-64 Years		Age ≥ 65 Years	
Seraphin	Placebo (*n* = 74)	Macitentan (*n* = 62)	Placebo (*n* = 131)^a^	Macitentan (*n* = 153)	Placebo (*n* = 44)	Macitentan (*n* = 27)
All-cause hospitalizations per year, mean (SD), *n*	0.9 (2.0)	0.8 (3.1)	0.8 (1.5)	0.4 (1.1)	1.7 (3.9)	0.3 (0.5)
GRIPHON	Placebo (*n* = 141)	Selexipag (*n* = 124)	Placebo (*n* = 333)	Selexipag (*n* = 351)	Placebo (*n* = 108)	Selexipag (*n* = 99)
All-cause hospitalizations per year, mean (SD), *n*	0.8 (1.5)	0.6 (1.5)	0.9 (2.2)	0.8 (2.1)	1.3 (2.9)	1.1 (2.3)

SD, standard deviation.

a One patient in the placebo group did not receive any study treatment and was not included in the pharmacoeconomic analysis.

**Table 5:** Evaluation of Treatment Effect in Prognostic Age Groups: Change in N-terminal Prohormone B-type Natriuretic Peptide in SERAPHIN and GRIPHON

Seraphin	Age < 35 Years		Age 35-64 Years		Age ≥ 65 Years	
	Placebo (*n* = 74)	Macitentan (*n* = 62)	Placebo (*n* = 132)	Macitentan (*n* = 153)	Placebo (*n* = 44)	Macitentan (*n* = 27)
Baseline, *n*	47	38	91	111	22	21
NT-proBNP, median (Q1, Q3), pg/mL	590.1 (432.4, 1068.2)	691.6 (521.0, 1171.7)	720.1 (527.3, 1242.1)	778.5 (547.4, 1383.8)	825.7 (616.6, 1380.9)	952.7 (672.2, 1730.8)
Change from baseline to month 6, *n*	47	38	91	111	22	21
NT-proBNP, median change (Q1, Q3), pg/mL	44.9 (−18.5, 283.4)	−38.1 (−234.1, 76.8)	64.1 (−47.1, 209.6)	−63.0 (−270.1, 54.3)	52.2 (−71.1, 195.3)	−15.3 (−317.5, 125.4)
*p*-value vs middle age group	0.94	0.93	Ref	Ref	0.91	0.89

GRIPHON	Age < 35 years		Age 35−64 years		Age ≥ 65 years	
	Placebo (*n* = 141)	Selexipag (*n* = 124)	Placebo (*n* = 333)	Selexipag (*n* = 351)	Placebo (*n* = 108)	Selexipag (*n* = 99)

Baseline, *n*	137	122	330	348	107	98
NT-proBNP, median (Q1, Q3), pg/mL	520.0 (159.0, 1802.0)	488.0 (152.0, 1498.0)	504.0 (179.0, 1563.0)	512.5 (166.5, 1394.0)	996.0 (290.0, 2397.0)	815.5 (347.0, 1761.0)
Change from baseline to month 6, *n*	114	103	255	286	80	71
NT-proBNP, median change (Q1, Q3), pg/mL	47.5 (−95.0, 264.0)	−12.0 (−155.0, 79.0)	10.0 (−84.0, 301.0)	−38.0 (−255.0, 87.0)	57.0 (−100.0, 567.5)	−56.0 (−239.0, 268.0)
*p*-value vs middle age group	0.90	0.66	Ref	Ref	0.51	0.78

NT-proBNP, N-terminal prohormone B-type natriuretic peptide; Q, quarter; Ref, reference value.

### Safety and tolerability

The overall incidence and pattern of serious adverse events (SAEs) were similar across the three age groups (Table 6). Across all age groups, the incidence of overall SAEs and SAEs of PAH worsening were consistently lower in the macitentan arm of SERAPHIN and the selexipag arm of GRIPHON versus placebo, except for the SAE preferred term ‘right ventricular failure’, which was more frequent with selexipag than placebo in the ≥ 65 years age group in GRIPHON (Table 6). The incidence of adverse events leading to macitentan discontinuation across age groups was 11.3% in the younger group, 9.8% in the middle group, and 14.8% in the older group. In the SERAPHIN placebo arm, discontinuation rates due to adverse events were 10.8% (younger), 9.2% (middle), and 25% (older). The incidence of adverse events leading to selexipag discontinuation was numerically greater among older patients (24.2%, younger; 31.1%, middle; 43.4%, older). However, this was also the case in the placebo arm of GRIPHON (31.7%, younger; 36.1%, middle; 46.7%, older).

**Table 6:** Serious Adverse Events and Adverse Events Leading to Treatment Discontinuation in SERAPHIN and GRIPHON by Age Group

Serious Adverse Events Occurring in > 2% of Patients in Any Group						
Seraphin^a^	Age < 35 Years		Age 35-64 Years		Age ≥ 65 Years	
	Placebo (*n* = 74)	Macitentan (*n* = 62)	Placebo (*n* = 131)	Macitentan (*n* = 153)	Placebo (*n* = 44)	Macitentan (*n* = 27)
At least one SAE, *n* (%)	41 (55.4)	30 (48.4)	70 (53.4)	67 (43.8)	26 (59.1)	12 (44.4)
PAH	17 (23.0)	7 (11.3)	30 (22.9)	21 (13.7)	9 (20.5)	4 (14.8)
Right ventricular failure	14 (18.9)	10 (16.1)	19 (14.5)	12 (7.8)	7 (15.9)	1 (3.7)
Pneumonia	3 (4.1)	1 (1.6)	3 (2.3)	2 (1.3)	2 (4.5)	1 (3.7)
Syncope	2 (2.7)	3 (4.8)	3 (2.3)	1 (0.7)	1 (2.3)	0
Sepsis	1 (1.4)	0	2 (1.5)	0	1 (2.3)	0
Hemoptysis	2 (2.7)	0	2 (1.5)	0	0	0
Atrial fibrillation	0	0	1 (0.8)	0	2 (4.5)	0
Pregnancy	2 (2.7)	0	0	0	0	0
Anemia	0	1 (1.6)	0	5 (3.3)	0	0
Atrial flutter	0	0	2 (1.5)	3 (2.)	0	1 (3.7)
Chest pain	0	0	0	2 (1.3)	0	1 (3.7)

GRIPHON	Age < 35 years		Age 35−64 years		Age ≥ 65 years	
	Placebo (*n* = 139)	Selexipag (*n* = 124)	Placebo (*n* = 332)	Selexipag (*n* = 351)	Placebo (*n* = 107)	Selexipag (*n* = 99)

At least one SAE, n (%)	66 (47.5)	47 (37.9)	145 (43.7)	153 (43.6)	61 (57.0)	52 (52.5)
PAH	39 (28.1)	21 (16.9)	71 (21.4)	52 (14.8)	17 (15.9)	10 (10.1)
Right ventricular failure	11 (7.9)	4 (3.2)	19 (5.7)	15 (4.3)	11 (10.3)	15 (15.2)
Pneumonia	5 (3.6)	2 (1.6)	13 (3.9)	11 (3.1)	7 (6.5)	4 (4.0)
Syncope	5 (3.6)	3 (2.4)	14 (4.2)	6 (1.7)	1 (0.9)	1 (1.0)
Dyspnea	5 (3.6)	4 (3.2)	4 (1.2)	7 (2.0)	4 (3.7)	6 (6.1)
Fall	0	0	2 (0.6)	1 (0.3)	4 (3.7)	2 (2.0)
Acute renal failure	1 (0.7)	0	1 (0.3)	2 (0.6)	4 (3.7)	4 (4.0)
Gastrointestinal hemorrhage	0	0	0	3 (0.9)	3 (2.8)	0
Upper respiratory tract infection	3 (2.2)	2 (1.6)	0	2 (0.6)	0	0
Atrial fibrillation	0	1 (0.8)	2 (0.6)	2 (0.6)	2 (1.9)	4 (4.0)
Anemia	2 (1.4)	0	1 (0.3)	2 (0.6)	0	3 (3.0)
Epistaxis	1 (0.7)	0	3 (0.9)	1 (0.3)	0	3 (3.0)

Adverse Events Leading to Treatment Discontinuation in > 1 Patient in Any Group						

SERAPHIN^a^	Age < 35 years		Age 35−64 years		Age ≥ 65 years	
	Placebo (*n* = 74)	Macitentan (*n* = 62)	Placebo (*n* = 131)	Macitentan (*n* = 153)	Placebo (*n* = 44)	Macitentan (*n* = 27)

Any AE leading to discontinuation, *n* (%)	8 (10.8)	7 (11.3)	12 (9.2)	15 (9.8)	11 (25.0)	4 (14.8)
PAH	2 (2.7)	0	5 (3.8)	1 (0.7)	3 (6.8)	3 (11.1)
Right ventricular failure	2 (2.7)	1 (1.6)	1 (0.8)	3 (2.0)	3 (6.8)	0
Headache	0	1 (1.6)	0	2 (1.3)	0	0

GRIPHON	Age < 35 years		Age 35−64 years		Age ≥ 65 years	
	Placebo (*n* = 139)	Selexipag (*n* = 124)	Placebo (*n* = 332)	Selexipag (*n* = 351)	Placebo (*n* = 107)	Selexipag (*n* = 99)

At least one AE leading to discontinuation, *n* (%)	44 (31.7)	30 (24.2)	120 (36.1)	109 (31.1)	50 (46.7)	43 (43.4)
PAH	30 (21.6)	13 (10.5)	76 (22.9)	47 (13.4)	29 (27.1)	18 (18.2)
Right ventricular failure	7 (5.0)	5 (4.0)	10 (3.0)	5 (1.4)	6 (5.6)	4 (4.0)
Dyspnea	5 (3.6)	2 (1.6)	3 (0.9)	4 (1.1)	2 (1.9)	1 (1.0)
Headache	0	2 (1.6)	4 (1.2)	12 (3.4)	0	5 (5.1)
Abnormal walking distance test	2 (1.4)	0	1 (0.3)	0	0	0
Acute renal failure	0	0	0	1 (0.3)	2 (1.9)	1 (1.0)
Respiratory failure	0	0	0	0	2 (1.9)	0
Diarrhea	0	2 (1.6)	0	7 (2.0)	0	4 (4.0)
Nausea	0	1 (0.8)	0	5 (1.4)	0	4 (4.0)
Pain in extremity	0	1 (0.8)	0	3 (0.9)	0	2 (2.0)
Sudden death	0	1 (0.8)	0	4 (1.1)	0	0
Myalgia	0	1 (0.8)	0	3 (0.9)	0	1 (1.0)
Abdominal pain	0	0	0	3 (0.9)	0	1 (1.0)
Dizziness	0	0	0	1 (0.3)	0	3 (3.0)
Asthenia	0	0	0	2 (0.6)	0	(1.0)

AE, adverse event; PAH, pulmonary arterial hypertension; SAE, serious adverse event.

a Note, one patient randomized to placebo in SERAPHIN did not receive study drug and was excluded from the safety analysis.

The overall incidence and pattern of treatment-emergent adverse events (TEAEs) were broadly similar across the three age groups and consistent with the findings in the overall population evaluated in SERAPHIN and GRIPHON (Table S2).^9^, ^10^ Across age groups, TEAEs related to PAH, peripheral edema, right ventricular failure, cough, and dizziness were generally less frequent with macitentan versus placebo. There was a trend for a numerically lower incidence of peripheral edema and dizziness in the youngest age group receiving macitentan (Table S2). TEAEs related to PAH, peripheral edema, and right ventricular failure were generally less frequent with selexipag than placebo. Consistent trends according to age group were not identified in the GRIPHON study (Table S2); however, across both studies sample sizes were small. Adverse events reported in the SERAPHIN trial as occurring at higher incidence with macitentan versus placebo (nasopharyngitis, headache, and anemia) were also consistently numerically higher across age groups. Adverse events reported significantly (p<0.001) more frequently with selexipag versus placebo in the GRIPHON trial (headache, diarrhea, nausea, vomiting, extremity pain, jaw pain, myalgia, and flushing) were also consistently reported more frequently across age groups, other than vomiting which was less frequently reported with selexipag than placebo in the oldest age group (Table S2).^9^, ^10^

## Discussion

This post-hoc, exploratory analysis identified age as an independent predictor of M/M risk among patients with PAH and suggests that patients aged < 35 years have a higher risk of M/M events than patients aged 35–64 years. Treatment with macitentan or selexipag reduced the risk of an M/M event versus placebo in all age groups by 28–56% with the greatest treatment effect in patients aged < 35 years, whose relative risk of M/M was halved.

As this is a post-hoc, exploratory analysis, the data should be interpreted with caution. Nevertheless, the findings provide a basis for further research. A notable finding from this analysis is the poor outcomes among the youngest age group evaluated (< 35 years) using the placebo groups from SERAPHIN and GRIPHON. Future analyses accounting for the baseline risk status of patients < 35 years of age will determine optimal risk of M/M calculators for this patient group. There was also some indication from SERAPHIN that placebo-treated patients aged ≥ 65 years had a higher risk of an M/M event than those aged 35–64 years. Although the numerical difference between these age groups was not statistically significant, this could be due to the small sample size in the older age group. Overall, it is possible that patients ≥ 65 years of age had a poorer prognosis for disease progression, even though a statistically greater risk of an M/M event could not be demonstrated.

There are a number of factors that could be driving the observed higher risk of M/M events in the younger (< 35 years) versus those aged 35–64 years. Like current registries, this analysis of randomized controlled trials identified similar trends in baseline characteristics among age groups, specifically that the youngest patients were more often in the lowest NYHA/WHO FC group, had fewer comorbidities, and had greater 6MWD, while also presenting with more severe impairment of hemodynamics, with higher mean pulmonary artery pressure and pulmonary vascular resistance.^2^, ^3^, ^12^ Clinically, these patients have the functional capacity to compensate for these less favorable hemodynamic parameters. However, once functional capacity reserves are depleted, these patients can deteriorate quickly, consistent with the findings from our analysis of an increased risk of M/M in the younger group (< 35 years). The overall safety and tolerability profile (in terms of incidence and pattern of SAEs) was similar across the three age groups and there were also similar rates of macitentan discontinuation across age groups.

Strengths of the present analysis are the randomized placebo-controlled design of the included studies, the identification of three distinct prognostic age groups rather than combining all patients aged < 65 years, and the use of clinical trial data for modern therapies to provide information about age-specific benefits of these treatments. This analysis is subject to several limitations. Although both SERAPHIN and GRIPHON provide large datasets for the analysis of age groups, the current analysis is post hoc and exploratory. As the analysis for primary endpoint of M/M in both SERAPHIN and GRIPHON did not include BMI or cardiovascular comorbidities as covariates per study design and pre-planned statistical analysis methods, the analyses reported here were not adjusted for these variables, which may confound the association between age and treatment analysis. There were also limited numbers of patients in the older and younger age groups, likely reflecting the more homogeneous patient populations that participate in clinical trials versus patients treated in real-world settings. However, it should be noted that GRIPHON included a relatively high proportion of patients aged ≥ 65 years (207 patients; 18% of the study population) compared with other randomized PAH clinical trials. It is also possible that differences in geographical regions and access to treatment impact treatment outcomes.

In conclusion, this post-hoc analysis of patients with PAH in the placebo arms of SERAPHIN and GRIPHON identified age < 35 years as a potential risk factor for M/M and suggested that age ≥ 65 years could be a factor in poorer prognosis for disease progression. Further analysis of these observations is warranted. A benefit with macitentan (in SERAPHIN) and selexipag (in GRIPHON) was observed across all age groups based on M/M events. Results from this post-hoc analysis are consistent with those reported in previous publications on the SERAPHIN and GRIPHON trials and included in the prescribing information for macitentan and selexipag.^8^, ^9^, ^13^, ^14^

## Author contributions

All authors contributed to the analysis concept. Carol Zhao and Joseph Yen analyzed the data. All authors contributed to data interpretation and manuscript writing. All authors approved the final version of the manuscript prior to submission.

## Footnotes

Appendix A Supplemental data associated with this article can be found in the online version at doi:10.1016/j.jhlto.2024.100197.
