# Supplementary material for: Effects of macitentan and selexipag across prognostic age groups in patients with pulmonary arterial hypertension
Source: JHLT Open. 2025 Jan 16;7:100197. doi: 10.1016/j.jhlto.2024.100197 (PMC11935431; doi:10.1016/j.jhlto.2024.100197)
Supplement: Supplementary file 1 — Supplemental material [file mmc1.pdf]

Supplemental material

Effects of macitentan and selexipag across prognostic age groups in patients with pulmonary arterial hypertension

Richard Channick, MD, Sarah Medrek, MD, Marion Delcroix, MD, Sean Gaine, MD, Pavel Jansa, MD, PhD, Irene Lang, MD, Vallerie McLaughlin, MD, Sanjay Mehta, MD, Tomas Pulido, MD, Bhagavatula Sastry, MD, Rogerio Souza, MD, PhD, Adam Torbicki, MD, Carol Zhao, MS, Peter Agron, PhD, and Olivier Sitbon, MD, PhD

Contents

	**Page**
**Figure S1** Identification of prognostic age groups. Likelihood of experiencing a morbidity/mortality event within 36 months in (A) SERAPHIN and (B) GRIPHON	2
**Table S1** Definitions of the Primary and Key Secondary Endpoints in SERAPHIN and GRIPHON	3
**Table S2** Treatment-Emergent Adverse Events Occurring in ≥ 15% of Patients in Any Age Group	5
**References**	8

Figure S1 Identification of prognostic age groups. Likelihood of experiencing a morbidity/mortality event within 36 months in (A) SERAPHIN and (B) GRIPHON. The dotted green (2-degree polynomial function) and blue (2-month moving average) lines represent the curve after smoothing functions were applied.

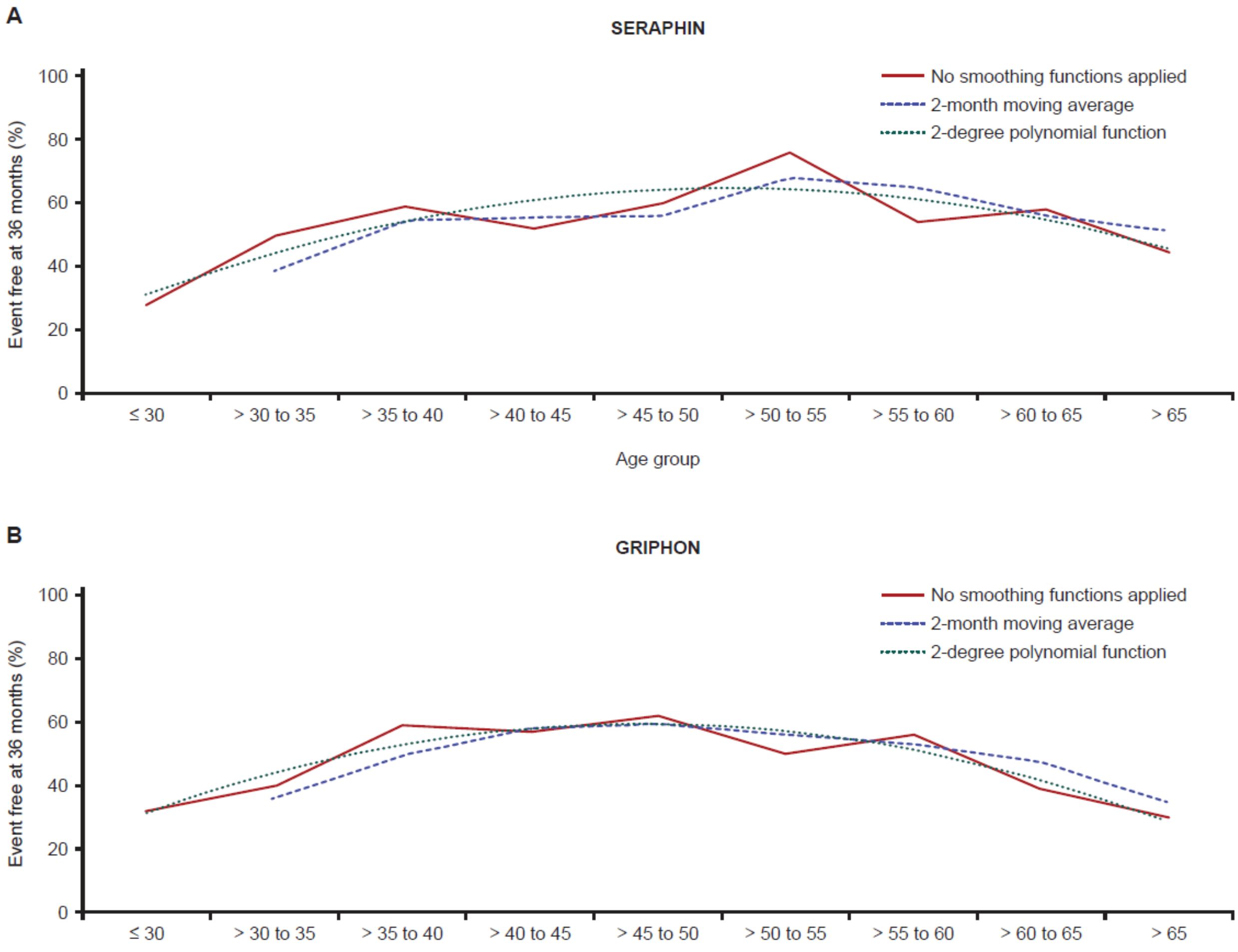

Table S1 Definitions of the Primary and Key Secondary Endpoints in SERAPHIN and GRIPHON[a]

SERAPHIN[1]		GRIPHON[2]	
**Primary endpoint: components of morbidity events**	**Key secondary endpoints**	**Primary endpoint: components of morbidity events**	**Key secondary endpoints**
Worsening of PAH defined as the occurrence of all 3 of the following:<br>• Decrease in 6MWD of ≥ 15% from baseline, confirmed by a second 6MWD test performed on a different day within 2 weeks<br>• Worsening of symptoms of PAH, defined as ≥ 1 of the following:<br>○ Change from baseline to a higher NYHA/WHO FC (or no change in patients who were in NYHA/WHO	• Change in 6MWD from baseline to month 6<br>• Patients (%) with NYHA/WHO FC improvement at month 6<br>• Death due to PAH or hospitalization for PAH up to the end of treatment<br>• Death from any cause up to the end of treatment and up to the end of the study	Disease progression, defined as both of the following:<br>• Decrease from baseline of ≥ 15% in the 6MWD (confirmed by means of a second test on a different day)<br>• Worsening in NYHA/WHO FC (for the patients with NYHA/WHO FC II or III at baseline) or the need for additional treatment of PAH (for the patients with NYHA/WHO FC III or IV at baseline)	• Change in 6MWD from baseline to week 26 (measured at trough levels of the study drug)<br>• Absence of NYHA/WHO FC worsening from baseline to week 26<br>• Death due to PAH or hospitalization for worsening of PAH up to the end of treatment period<br>• Death from any cause up to the end of the study<br>• Change in NT-proBNP from baseline to week 26 (exploratory endpoint)

FC IV at baseline)<br>o Appearance or worsening of signs of right heart failure that did not respond to oral diuretic therapy<br>• The need for additional treatment for PAH			
Initiation of treatment with IV or SC prostanoids		Initiation of parenteral prostanoid therapy or long-term oxygen therapy[b]	
Lung transplantation		Balloon atrial septostomy[b]	
Atrial septostomy		Hospitalization[b]	

6MWD, 6-minute walk distance; IV, intravenous; NT-proBNP, N-terminal prohormone B-type natriuretic peptide; PAH, pulmonary arterial hypertension; SC, subcutaneous; NYHA/WHO FC, New York Heart Association/World Health Organization functional class.

[a]Adapted from: McLaughlin VV, Hoeper MM, Channick RN, et al. Pulmonary arterial hypertension-related morbidity is prognostic for mortality. J Am Coll Cardiol 2018;71:752-63.

[b]For worsening PAH.

Table S2 Treatment-Emergent Adverse Events Occurring in ≥ 15% of Patients in Any Age Group

**SERAPHIN[a] (TEAEs occurring ≤ 28 days after treatment end)**	**Age < 35 years**		**Age 35-64 years**		**Age ≥ 65 years**	
	**Placebo (*n* = 74)**	**Macitentan (*n* = 62)**	**Placebo (*n* = 131)**	**Macitentan (*n* = 153)**	**Placebo (*n* = 44)**	**Macitentan (*n* = 27)**
At least one TEAE, *n* (%)	70 (94.6)	58 (93.5)	126 (96.2)	144 (94.1)	44 (100.0)	27 (100.0)
PAH	26 (35.1)	13 (21.0)	46 (35.1)	31 (20.3)	15 (34.1)	9 (33.3)
Edema peripheral	12 (16.2)	5 (8.1)	25 (19.1)	32 (20.9)	8 (18.2)	7 (25.9)
Right ventricular failure	22 (29.7)	13 (21.0)	24 (18.3)	16 (10.5)	10 (22.7)	3 (11.1)
Upper respiratory tract infection	9 (12.2)	7 (11.3)	21 (16.0)	26 (17.0)	3 (6.8)	4 (14.8)
Nasopharyngitis	5 (6.8)	7 (11.3)	17 (13.0)	22 (14.4)	4 (9.1)	5 (18.5)
Headache	6 (8.1)	6 (9.7)	13 (9.9)	24 (15.7)	3 (6.8)	3 (11.1)
Dizziness	10 (13.5)	2 (3.2)	11 (8.4)	19 (12.4)	6 (13.6)	5 (18.5)
Cough	8 (10.8)	5 (8.1)	14 (10.7)	14 (9.2)	8 (18.2)	2 (7.4)
Anemia	4 (5.4)	6 (9.7)	3 (2.3)	23 (15.0)	1 (2.3)	3 (11.1)
Urinary tract infection	1 (1.4)	4 (6.5)	6 (4.6)	14 (9.2)	7 (15.9)	3 (11.1)

Back pain	2 (2.7)	3 (4.8)	11 (8.4)	2 (1.3)	8 (18.2)	4 (14.8)
**GRIPHON (TEAEs occurring ≤ 7 days after treatment end)**	**Age < 35 years**		**Age 35-64 years**		**Age ≥ 65 years**	
	**Placebo (*n* = 139)**	**Selexipag (*n* = 124)**	**Placebo (*n* = 332)**	**Selexipag (*n* = 351)**	**Placebo (*n* = 107)**	**Selexipag (*n* = 99)**
At least one TEAE, *n* (%)	135 (97.1)	121 (97.6)	322 (97.0)	344 (98.0)	103 (96.3)	99 (100.0)
Headache	39 (28.1)	84 (67.7)	121 (36.4)	230 (65.5)	30 (28.0)	60 (60.6)
Diarrhea	21 (15.1)	46 (37.1)	67 (20.2)	149 (42.5)	22 (20.6)	49 (49.5)
PAH	58 (41.7)	28 (22.6)	110 (33.1)	75 (21.4)	38 (35.5)	23 (23.2)
Nausea	26 (18.7)	52 (41.9)	62 (18.7)	111 (31.6)	19 (17.8)	30 (30.3)
Dyspnea	27 (19.4)	18 (14.5)	58 (17.5)	54 (15.4)	36 (33.6)	20 (20.2)
Edema peripheral	21 (15.1)	16 (12.9)	62 (18.7)	49 (14.0)	21 (19.6)	15 (15.2)
Pain in jaw	6 (4.3)	31 (25.0)	23 (6.9)	98 (27.9)	7 (6.5)	19 (19.2)
Dizziness	20 (14.4)	21 (16.9)	48 (14.5)	49 (14.0)	17 (15.9)	16 (16.2)
Upper respiratory tract infection	24 (17.3)	31 (25.0)	44 (13.3)	36 (10.3)	12 (11.2)	8 (8.1)
Vomiting	13 (9.4)	38 (30.6)	28 (8.4)	63 (17.9)	8 (7.5)	3 (3.0)
Pain in extremity	4 (2.9)	9 (7.3)	29 (8.7)	60 (17.1)	13 (12.1)	28 (28.3)

Nasopharyngitis	12 (8.6)	15 (12.1)	45 (13.6)	45 (12.8)	6 (5.6)	15 (15.2)
Myalgia	7 (5.0)	14 (11.3)	22 (6.6)	62 (17.7)	5 (4.7)	16 (16.2)
Right ventricular failure	13 (9.4)	10 (8.1)	32 (9.6)	18 (5.1)	13 (12.1)	18 (18.2)
Flushing	5 (3.6)	10 (8.1)	19 (5.7)	45 (12.8)	5 (4.7)	15 (15.2)

PAH, pulmonary arterial hypertension; TEAE, treatment-emergent adverse event.

[a]Note: one patient randomized to placebo in SERAPHIN did not receive study drug and was excluded from the safety analysis.

References

1. Pulido T, Adzerikho I, Channick RN, et al. Macitentan and morbidity and mortality in pulmonary arterial hypertension. N Engl J Med 2013;369:809-18.

2. Sitbon O, Channick R, Chin KM, et al. Selexipag for the treatment of pulmonary arterial hypertension. N Engl J Med 2015;373:2522-33.
